# Phytochemical Analysis and Evaluation of Biological Activity of *Lawsonia inermis* Seeds Related to Alzheimer's Disease

**DOI:** 10.1155/2021/5965061

**Published:** 2021-07-19

**Authors:** Majid Balaei-Kahnamoei, Mina Saeedi, Arezoo Rastegari, Mohammad Reza Shams Ardekani, Tahmineh Akbarzadeh, Mahnaz Khanavi

**Affiliations:** ^1^Department of Pharmacognosy, Faculty of Pharmacy, Tehran University of Medical Sciences, Tehran, Iran; ^2^Medicinal Plants Research Center, Faculty of Pharmacy, Tehran University of Medical Sciences, Tehran, Iran; ^3^Persian Medicine and Pharmacy Research Center, Tehran University of Medical Sciences, Tehran, Iran; ^4^Department of Medicinal Chemistry, Faculty of Pharmacy, Tehran University of Medical Sciences, Tehran, Iran; ^5^Faculty of Land and Food Systems, University of British Columbia, Vancouver, British Columbia, Canada

## Abstract

Using *Lawsonia inermis* L. (henna) seeds has been frequently recommended for the improvement of memory in Iranian Traditional Medicine (ITM). In this respect, different fractions of the plant were prepared and evaluated for their *in vitro* biological assays related to Alzheimer's disease (AD), including acetylcholinesterase (AChE) and butyrylcholinesterase (BChE) inhibitory activity as well as metal chelating ability and DPPH antioxidant activity. The dichloromethane and ethyl acetate fractions were able to inhibit the BChE selectively with IC_50_ values of 113.47 and 124.90 *μ*g/mL, respectively, compared with donepezil as the reference drug (IC_50_ = 1.52 *μ*g/mL). However, all fractions were inactive toward AChE. Phytochemical analysis of the dichloromethane fraction indicated the presence of *β*-sitosterol (**1**), 3-*O-β-*acetyloleanolic acid (**2**), 3-*O*-(*Z*)-coumaroyl oleanolic acid (**3**), betulinic acid (**4**), and oleanolic acid (**5**). The inhibitory activity of isolated compounds was also evaluated toward AChE and BChE. Among them, compounds **2** and **5** showed potent inhibitory activity toward BChE with IC_50_ values of 77.13 and 72.20 *μ*M, respectively. However, all compounds were inactive toward AChE. Moreover, molecular docking study confirmed desired interactions between those compounds and the BChE active site. The ability of fractions and compounds to chelate biometals (Cu^2+^, Fe^2+^, and Zn^2+^) was also investigated. Finally, DPPH antioxidant assay revealed that the ethyl acetate (IC_50_ = 3.08 *μ*g/mL) and methanol (IC_50_ = 3.64 *μ*g/mL) fractions possessed excellent antioxidant activity in comparison to BHA as the positive control (IC_50_ = 3.79 *μ*g/mL).

## 1. Introduction

Alzheimer's disease (AD) is characterized as the most common neurodegenerative disease leading to a gradual decrease in memory, cognitive disorders, psychological and behavioral disturbances, and serious problems in daily activities. Also, it accounts for more than 70% of dementia cases in elderly people worldwide [1]. The number of patients with AD is predicted to be triple in 2050 [2]. Not only the increasing number of patients with AD in both developed and developing countries, but also the economic burden of disease has encouraged the researchers to develop efficient anti-AD drugs [3].

AD is the consequence of multiple etiological factors including genetics, environment, and lifestyle [4]. The exact origin of AD is not clear and different factors including reduced levels of acetylcholine (ACh) in the brain [5], intracellular hyperphosphorylation of tau protein and formation of neurofibrillary tangles (NFTs) [6], accelerated aggregation of *β*-amyloid peptides [7], dyshomeostasis and miscompartmentalization of the biometal ions (Fe^2+^, Cu^2+^, and Zn^2+^) [8], calcium overload and mitochondrial disruption [9], and oxidative stress due to the generation of reactive oxygen species (ROS) [10] play important roles in the onset and progression of the disease.

Now, it is clear that ACh levels in the hippocampus and cortex are crucial for the regulation of memory, attention, learning, and motivation [11]. Cholinesterases (ChEs) including acetylcholinesterase (AChE) and butyrylcholinesterase (BChE) are two cholinergic enzymes involved in the hydrolysis of ACh into choline and acetic acid to terminate synaptic transmission in the brain. In spite of the fact that physiological function of AChE at cholinergic synapses is well articulated, the function of BChE has still not been clarified [12]. In a healthy brain, both activity and concentration of AChE are more dominant than those of BChE. BChE has shown low activity in hydrolytic reactions and mainly distributed in plasma and tissues. However, the supportive role of this enzyme in synaptic transmission has been proven [13, 14]. During the progression of AD, hydrolysis of ACh is performed by BChE as the level of AChE shows a steady decline to 50%, that of BChE reaches 900%, and the BChE/AChE ratio also increases significantly [15]. It has been demonstrated that BChE regulates ghrelin levels in male BALB/c mice, which is responsible for emotional and social behaviors such as aggression [16]. Moreover, BChE activity was found to be very high in the hippocampus of patients with AD, the tissue which is strongly associated with cognitive functions [17]. All these indicate that the inhibition of AChE alone does not afford a proper planning process for the treatment of AD and the inhibition of BChE should also be considered.

Currently, there is no definite cure for AD, and most available drugs approved by FDA, such as donepezil, galantamine, and rivastigmine, are ChE inhibitors that improve cognitive disorders and the disease symptoms [18]. In this respect, development of ChE inhibitors is still in demand and it should be noted that selective anti-AChE activity can be achieved by small molecules, while selective anti-BChE activity is possible by bulky ligands since BChE has displayed low substrate specificity [19]. Another point that highlights the importance of the inhibition of ChEs is their noncholinergic role especially that of BChE in the deposition of *β*A plaques [20].

The development of anti-AD drugs is directly affected by the multifactorial nature of the disease; hence, medicinal plants possessing a wide range of phytochemicals are the potent candidates for the AD drug discovery [21]. *Lawsonia inermis* L., commonly known as “henna” [22], belongs to Lythraceae family and is a native of Southwest Asia and North Africa [23]. *L. inermis* is known for its valuable applications in the cosmetic industry [24] and traditional therapeutic properties such as edema, bronchitis, rheumatism, smallpox, spermatorrhoea, menstrual disorders, and hemorrhoids [25]. Antidiarrheal, anti-inflammatory, analgesic, antipyretic, antibacterial especially Gram-positive bacteria, and antifungal properties against *Trichophyton*, *Sporotrichum*, and *Cryptococcus* have been recorded in the literature [26]. According to Iranian Traditional Medicine (ITM), consuming henna seeds with honey and tragacanth strengthens and improves memory [27]. Herein, in continuation of our efforts on development of herbal anti-AD agents [28–32], we selected *L. inermis* seeds to evaluate different biological activities related to AD including *in vitro* ChEs inhibitory activity as well as metal chelating ability (copper, iron, and zinc ions) and DPPH antioxidant activity. Also, according to the efficacy of dichloromethane fraction, phytochemical analysis led to the isolation and identification of five compounds that have not been previously reported for henna seeds.

## 2. Materials and Methods

### 2.1. General Experimental Procedures

Melting points were determined on a Kofler hot stage apparatus and are uncorrected. NMR spectra were recorded on an Avance III spectrometer (Bruker) operating at 400.20 MHz for ^1^H-NMR and 100.63 MHz for ^13^C-NMR as well as 2D HMBC, COSY, and HSQC experiments. Silica gel for column chromatography (70–230 and 230–400 mesh) and precoated silica gel F_254_ (20 × 20 cm) plates for TLC were purchased from Merck. TLC plates were visualized under UV light (254 and 366 nm) as well as spraying anisaldehyde-sulfuric acid, followed by heating at 150°C.

### 2.2. Chemical and Reagents

Acetylcholinesterase (AChE, E.C. 3.1.1.7, Type V-S, lyophilized powder, from electric eel, 1000 units), butyrylcholinesterase (BChE, E.C. 3.1.1.8, from equine serum), acetylthiocholine iodide (ATCI), and 5,5-dithiobis-2-nitrobenzoic acid (DTNB) were purchased from Sigma-Aldrich. Potassium dihydrogen phosphate, dipotassium hydrogen phosphate, potassium hydroxide, and sodium hydrogen carbonate were obtained from Fluka. Solvents for the extraction and column chromatography (CC) were of technical grade and redistilled before use. Deuterated solvents (100 atom % D) were obtained from Sigma-Aldrich.

### 2.3. Plant Material

Fresh and healthy fruits of *Lawsonia inermis* L. were collected from the southern coast of Iran, Hormozgan Province, Bandar Abbas City, and the taxonomic identities of the plant were authenticated by Doctor Ajani, a taxonomist at the Research Institute of Forests and Rangelands, and deposited in the Herbarium of Faculty of Pharmacy, Tehran University of Medical Sciences (voucher specimen no. 7028-TEH).

### 2.4. In Vitro AChE/BChE Inhibition Assay

*In vitro* anti-ChEI activity was performed against acetylcholinesterase (AChE, E.C. 3.1.1.7, Type V-S, lyophilized powder, from electric eel, 1000 units) and butyrylcholinesterase (BChE, E.C. 3.1.1.8, from equine serum) using the modified Ellman's method [33]. To obtain acceptable enzyme inhibitory activity (20–80%), the stock solutions of the fractions (10 mg/mL) and compounds (1 mg/mL) were prepared in DMSO and were diluted with a mixture of DMSO and methanol to achieve four different final concentrations of the fractions (63.5, 125, 250, 500 *μ*g/mL) and compounds (1, 10, 20, 40 *μ*g/mL), while obtaining the final ratio of 50/50 DMSO/methanol. Each well consisted of 50 *μ*L potassium phosphate buffer (KH_2_PO_4_/K_2_HPO_4_, 0.1 M, pH 8), 25 *μ*L prepared sample as described above, and AChE enzyme (25 *μ*L) with a final concentration of 0.22 units/mL in the buffer. They were preincubated for 15 min at room temperature, and then, 125 *μ*L DTNB (3 mM in buffer) was added to the mixture. Following the addition of 25 *μ*L substrate ATCI (3 mM in water), changes in the absorbance were spectrometrically measured at 405 nm using a UV Unico Double Beam spectrophotometer. In parallel, a blank containing all components without enzyme was used in order to account for the nonenzymatic reaction. A negative control was also used under the same conditions without inhibitor, and donepezil was used as the positive control. The IC_50_ values were determined graphically from log concentration vs. % of inhibition curves. All experiments were performed in triplicate. BChE inhibition assay was performed in the same method.

### 2.5. Metal Ion Chelating Ability

All solutions used in metal chelating study were prepared in methanol. The solutions of Fe^2+^, Cu^2+^, and Zn^2+^ ions were obtained from FeSO_4_.7H_2_O, CuCl_2_.2H_2_O, and ZnCl_2_, respectively. To investigate the biometal chelating ability, a solution of compound or fraction at distinct concentration (1 mL) was mixed with the metal solution (1 mL) at the same concentration in a 1 cm quartz cuvette, and the mixture was left at room temperature for 30 min. Then, the absorbance of the solution was read in the wavelength range of 260–500 nm [34].

### 2.6. Antioxidant Activity by DPPH Test

Several concentrations of the test fraction in MeOH were prepared. Aliquots of different concentrations of the fraction (1 mL) were added to the DPPH methanolic solution (1.0 mL, 0.1 mM), and the mixtures were shaken vigorously and left for 30 min at room temperature in the dark. The absorbance was then measured at 517 nm using a UV⁄visible spectrophotometer. The percent scavenging activity was calculated using the following formula:

(1) $$\begin{array}{c} \text{inhibition}\left(\text{\%}\right)=\left[1-\frac{A\;\left(\text{sample}\right)\;-A\;\left(\text{blank}\right)\;}{A\;\left(\text{control}\right)}\right]\times 100, \end{array}$$

where A (sample) is the absorbance of the fractions, A (blank) is the maximum concentration of samples without DPPH, and A (control) is the absorbance of the DPPH.

In the same procedure, butylated hydroxyanisole (BHA) was used as the positive control. The DPPH radical scavenging activity of the fractions was expressed as IC_50_ (*μ*g/mL), which is obtained from linear regression plot between concentrations of the test fractions and percent inhibitions.

### 2.7. Molecular Docking Study

A molecular docking study was carried out using the Autodock 4.2.6 program for compounds **2** and **5**. The crystal structure of apo BChE (PDB ID: 4TPK) was retrieved from the Protein Data Bank (http://www.rcsb.org). The 3D structure of donepezil (positive control) was obtained from the DrugBank database (https://go.drugbank.com). The 2D structures of the desired compounds were drawn on ChemDraw Professional 16.0, and 3D structures were generated using Chem3D suite, saved in .pdb format, and converted to .pdbqt format coordinate by AutoDockTools (ADT). Moreover, for preparation of the pdbqt form of the enzymes, polar hydrogen atoms were added to amino acid residues and Kollman charges were assigned to all atoms using ADT, and the obtained enzyme .pdbqt was used as an input for the Autogrid program. In Autogrid for each atom type, the inhibitor's maps were calculated with 0.375 A° spacing between grid points, and the center of the grid box was placed *x* = 1.176, *y* = 11.017, and *z* = 11.175 for BChE. The dimensions of the active site box were set at 40 × 40 × 40 × ^A°^ for BChE [35]. Flexible ligand dockings were accomplished for the selected compounds. The docking was carried out with 100 runs using the Lamarckian genetic algorithm (LGA). Other parameters were accepted as default. The calculated geometries were ranked in terms of free energy of binding and the best pose was selected for further analysis. Molecular visualizations were performed by Discovery Studio 4.0 client software.

### 2.8. Extraction and Isolation

After drying the fruits of the plant in the shade and away from the sun, the seeds, a brown cone, were carefully separated from the capsule-like fruits, and then, the skin of the fruits and other plant organs were removed. Next, 400 g seeds were milled using a laboratory-scale mill and extracted with methanol (MeOH) 100% (5 × 2 L) for 72 h at room temperature. The collected extract was concentrated under vacuum at 40°C using a rotary evaporator, and finally, 84.20 g dry extract was obtained, yielding 21.05%. This extract was subjected to silica gel-column chromatography (CC) (400 g, 70–230 mesh, 10 × 30 cm) and washed with petroleum ether (PE), dichloromethane (DCM), ethyl acetate (EtOAc), and MeOH (4 L per solvent), respectively.

The DCM fraction was loaded onto a silica gel vacuum liquid chromatography (VLC) (200 g, 70–230 mesh, 4.5 × 70 cm), eluted with a gradient of the PE : DCM, and then DCM : acetone (ACE). Totally, 13 fractions (F1–F13) were combined with the aid of TLC analysis (bands were detected on TLC under UV (254 nm and 360 nm) and spraying anisaldehyde-sulfuric acid, followed by heating at 150°C.

Fraction F4 (250 mg, eluted with DCM 100%) was applied on silica gel CC (60 g, 70–230 mesh, 2 × 70 cm) and eluted with a gradient of the PE : ACE (95 : 5 to 80 : 20) to afford six subfractions (SFs. 4a-4f). Subfraction 4d was recrystallized from chloroform to give compound **1** (4 mg). Fraction F5 (500 mg, eluted with DCM : ACE (99 : 1)) was subjected to another silica gel CC (60 g, 70–230 mesh, 2 × 70 cm), eluted with a gradient of the PE : ACE (90 : 10 to 65 : 35) to afford three subfractions (SFs. 5a–5c). Subfraction 5b was further separated on silica gel CC (45 mg, 230–400 mesh, 2 × 70 cm), eluted with PE : ACE (85 : 15) to give compound **2** (10 mg). Fraction F8 (600 mg, eluted with DCM : ACE (96 : 4)) was separated over a silica gel CC (40 g, 230–400 mesh, 1.5 × 70 cm) with a gradient mixture of PE : ACE (80 : 20 to 0 : 100) as eluent, to afford thirteen subfractions (SFs. 8a–8m). Subfraction 8f was triturated with EtOAc to give an insoluble solid, which was recrystallized from MeOH to afford compound **3** (8 mg). Subfraction 8d was loaded onto silica gel CC (65 mg, 230–400 mesh, 2 × 100 cm), eluted with a gradient mixture of *n*-hexane : ethyl acetate (80 : 20) to give five subfractions (SFs. 8d1–8d5). Two subfractions, 8d1 and 8d3, were recrystallized from MeOH to afford compound **4** (7 mg) and compound **5** (8 mg), respectively.

## 3. Results and Discussion

### 3.1. Isolated Compounds from Henna Seeds

**Figure 1:**
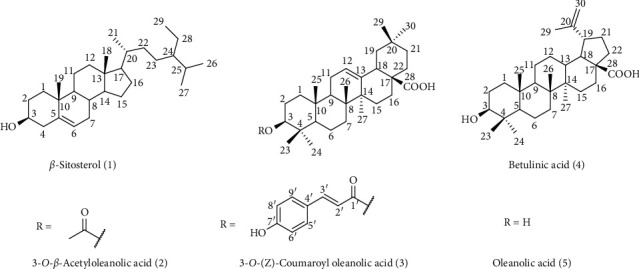
The structure of isolated compounds (**1**–**5**) from *L. inermis* seeds.

Phytochemical analysis of dichloromethane fraction of *L. inermis* seeds was conducted using silica gel column chromatography to isolate and characterize five compounds **1**–**5** for the first time for henna seeds (Figure 1). All data from the characterization of compounds **1**–**5** were compared with those reported in the literature [36–40].

*β*-Sitosterol (**1**): colorless needles (4 mg), mp: 134–137°C. ^1^H NMR (400 MHz, CDCl_3_) *δ* = 5.35 (1H, *d*, *J* = 5.3 Hz, H-6), 3.52 (1H, *m*, H-3), 1.01 (3H, s, Me-19), 0.92 (3H, *d*, *J* = 6.6 Hz, Me-21), 0.79–0.87 (9H, *m*, Me-29, Me-26, Me-27), 0.68 (3H, s, Me-18). ^13^C NMR (125 MHz, CDCl_3_, based on DEPT, HMQC and HMBC experiments; see Table 1) [36].

3-*O-β*-Acetyloleanolic acid (**2**): colorless crystal; m.p. >250°C. ^1^H NMR (400 MHz, CDCl_3_) *δ* = 5.27 (1H, *m*, H-12), 4.49 (1H, *m*, H-3), 2.82 (1H, dd, *J* = 13.9, 4.6 Hz, H-18), 2.05 (3H, *s*, 3-OAc), 0.74, 0.85, 0.86, 0.90, 0.93, 0.94, 1.12 (21H, *s*, CH_3_ × 7). ^13^C NMR (125 MHz, CDCl_3_, based on DEPT, HMQC and HMBC experiments; see Table 1) [37].

3-*O*-(*Z*)-Coumaroyl oleanolic acid (**3**): white powder, m.p. >250°C. ^1^H NMR (400 MHz, DMSO-*d*_6_): *δ* = 7.61 (2H, *d*, *J* = 8.5 Hz, H-5′ and H-9′), 7.60 (1H, *d*, *J* = 16.1 Hz, H-3′), 6.86 (2H, *d*, *J* = 8.5 Hz, H-6′ and H-8′), 6.43 (1H, *d*, *J* = 16.1 Hz, H-2′), 5.24 (1H, bs, H-12), 4.59 (1H, dd, *J* = 11.4, 4.3 Hz, H-3), 1.19, 0.99, 0.97, 0.95, 0.95, 0.92, 0.81 (21H, *s*, CH_3_ × 7). ^13^C NMR (125 MHz, CDCl_3_, based on DEPT, HMQC, and HMBC experiments); see Table 1 [38].

Betulinic acid (**4**): white amorphous powder, m.p. >250°C. ^1^H NMR (400 MHz, DMSO-d_6_) *δ* = 12.07 (1H, *s*, -COOH), 4.69 (1H, *d*, *J* = 1.8 Hz, H29a), 4.78 (1H, *t*, *J* = 1.8 Hz, H-29b), 4.28 (1 H, *d*, *J* = 5.1, OH-C_3_), 2.96 (2H, *m*, H-3 and H-19), 1.65, 0.94, 0.88, 0.88, 0.77, 0.65 (21H, *s*, CH_3_ × 7). ^13^C NMR (125 MHz, CDCl_3_, based on DEPT, HMQC, and HMBC experiments; see Table 1) [39].

Oleanolic acid (**5**): white amorphous powder, m.p. >250°C. ^1^H NMR (400 MHz, CDCl_3_) *δ* = 12.05 (1H, bs, HOO-C_28_), 5.19 (1H, *m*, H-12), 4.32 (1H, *d*, *J* = 5.1 Hz, OH-C_3_), 3.03 (1H, *m*, H-3), 2.77 (1H, dd, *J* = 13.9, 4.6 Hz, H-18), 1.84 (1H, dd, *J* = 9.0, 3.6 Hz, H-9), 1.26, 1.12, 0.92, 0.90, 0.88, 0.74, 0.70 (21H, *s*, CH_3_ × 7), 0.69 (1H, bs, H-5). ^13^C NMR (125 MHz, CDCl_3_, based on DEPT, HMQC, and HMBC experiments; see Table 1) [40].

### 3.2. Anticholinesterase Inhibitory Activity 

As anticholinesterases are still prescribed for the treatment of symptoms and cognitive impairment in patients with AD [41], potency of *L. inermis* seeds absorbed our attention due to recommendations in ITM. In this respect, all fractions and isolated compounds were screened for their *in vitro* AChE and BChE inhibitory activity using a 96-well microplate reader according to the modified Ellman's method [42] comparing with donepezil as the reference drug (IC_50_ = 1.52 *μ*g/mL) (Table 2).

It should be noted that all fractions and compounds **1**–**5** demonstrated no AChEI activity; however, results related to the selective anti-BChE activity were worth considering. As can be seen in Table 2, DCM and EtOAc fractions were the most potent and selective inhibitors of BChE with IC_50_ values of 113.47 ± 1.25 and 124.90 ± 1.15 *μ*g/mL, respectively, whereas the other fractions were not active. In the case of isolated compounds, compounds **2** and **5** depicted selective BChE inhibitory activity with IC_50_ values of 77.13 ± 0.01 and 72.20 ± 0.42 *μ*M, respectively.

The compensatory role of BChE in late-stage or advanced AD has been fully proven and the enzyme is responsible for the reduction of the ACh levels in the brain. Also, BChE plays an important role in the transformation of nonfibrillary to fibrillar A*β* plaques and senile plaques. It seems that inhibition of BChE can be considered as a therapeutic target in the treatment of AD [43]. In this respect, medicinal plants and natural products have been found to possess desired anticholinesterase activity. Plant-derived alkaloids, such as physostigmine, galantamine, sanguinine, and huperzine A, have shown significant inhibitory activity against acetylcholinesterase [44]. Terpenes, sterols, flavonoids, and glycosides have also been reported to possess anti-ChE activity [45]. Among fifteen compounds isolated from *Gelsemium elegans* and *Aglaia odorata*, paeonol and hydroperoxy-24-vinylcholesterol exhibited selective BChE inhibitory activity [46]. Also, sixteen lanostane triterpenes obtained from fruiting bodies of *Ganoderma lucidum* were selective inhibitors of AChE [44]. All these findings revealed that medicinal plants are the most credible source of ChE inhibitors.

The effect of *L. inermis* has been investigated from different points of view. Amat-ur-Rasool et al. reported that the methanolic extract of henna leaves had strong *in vitro* AChEI and BChEI activity with IC_50_ values of 0.33 and 0.41 mg/mL, respectively [47]. Also, study of the ethanolic extract of henna leaves against scopolamine-induced memory impairment in Swiss albino mice revealed that using the extract at the doses of 200 mg/kg and 400 mg/kg for 7 days inhibited AChE significantly as compared with the control group [48]. As reported by Mir et al., *in vivo* investigation of the ethyl acetate and chloroform extracts of *L. inermis* leaves at a dose of 25 mg/kg indicated their memory-enhancing potentials using two methods including “without inducing amnesia” and “induction of amnesia” by administration of diazepam as well as the cognitive improvements using behavioral models including elevated plus maze (EPM) and the passive shock avoidance (PSA) paradigm [49].

### 3.3. Metal Ion Chelating Activity

Redox-active metals such as Fe^2+^, Zn^2+^, and Cu^2+^ can increase the toxicity of A*β* plaques by forming coordinate bonds. Also, excessive accumulation of metal ions and interaction with *β*-amyloid plaques leads to oxidative stress due to an increased formation of reactive oxygen species (ROS). It seems that the biometal chelation hypothesis can be considered as a strong therapeutic tool in the treatment of AD [28]. Thus, the metal chelating ability of all fractions and isolated compounds **1**–**5** was investigated. For this purpose, the UV-visible absorption spectra were recorded in the wavelength range of 260–500 nm and compared with those obtained from the treated solution of fractions or compounds with Fe^2+^, Zn^2+^, and Cu^2+^ ions with final concentrations of 20 *μ*g/mL and 20 *μ*M, respectively. Those findings indicated the desired capability of phytochemicals present in the fractions to form complexes with biometals.

**Figure 2:**
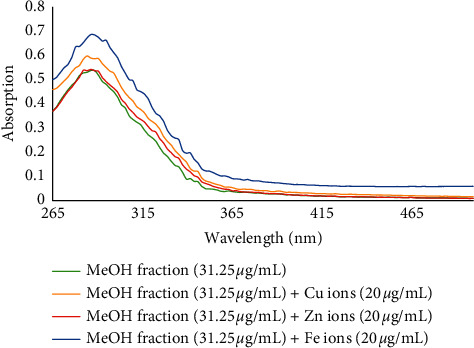
The absorbance changes of MeOH fraction alone and in the presence of Zn^2+^, Fe^2+^, and Cu^2+^ ions.

The MeOH fraction was able to chelate Cu^2+^ ions more strongly than Fe^2+^ and Zn^2+^ ions. The absorbance peak of MeOH fraction (final concentration of 31.25 *μ*g/mL) at 286.4 nm changed to 284.3 nm after 30 min interaction with Cu^2+^ ions (Figure 2).

**Figure 3:**
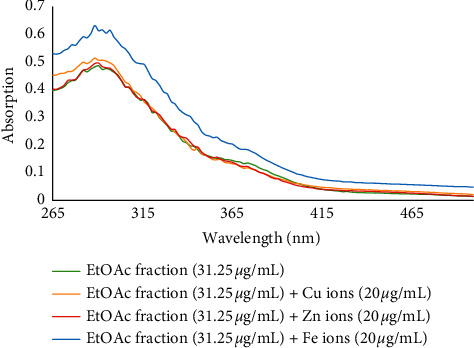
The absorbance changes of EtOAc fraction alone and in the presence of Zn^2+^, Fe^2+^, and Cu^2+^ ions.

The EtOAc fraction (final concentration of 31.25 *μ*g/mL) showed three absorption peaks at 294.9, 290.7, and 282.1 nm. It was capable of Cu^2+^ chelating as a peak at 288.5 nm appeared after interaction with Cu^2+^ ions. The interaction with Fe^2+^ ions led to a change in the absorption wavelength (288.5 and 297.1 nm). However, the fraction could not chelate Zn^2+^ ions (Figure 3).

**Figure 4:**
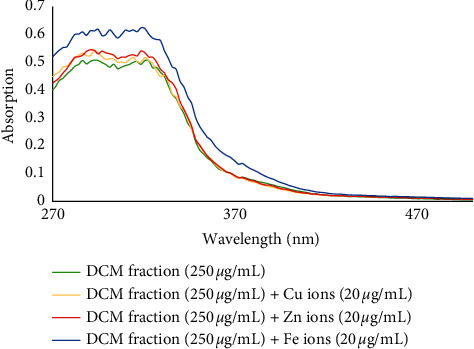
The absorbance changes of DCM fraction alone and in the presence of Zn^2+^, Fe^2+^, and Cu^2+^ ions.

The UV spectrum of DCM fraction (final concentration of 250 *μ*g/mL) showed several absorption peaks at 326.9, 320.5, 316.3, 303.5, 292.8, and 286.4 nm. Interaction of the fraction with Cu^2+^ ions led to the appearance of new peaks at 307.7 nm, a red shift from 326.9 to 331.2 nm, and a blue shift from 316.3 nm to 314.1 nm. In the case of interaction with Zn^2+^ ions, a blue shift from 303.5 to 297.1 was clear. Interaction of the sample with Fe^2+^ ions demonstrated absorption peaks at 318.4, 314.1, 309.9, 301.3, 294.9, 290.7, and 286.4 nm (Figure 4).

**Figure 5:**
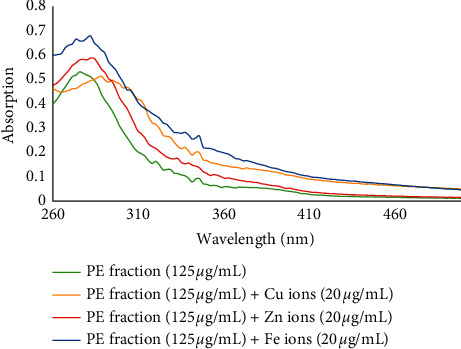
The absorbance changes of PE fraction alone and in the presence of Zn^2+^, Fe^2+^, and Cu^2+^ ions.

The PE fraction (final concentration of 125 *μ*g/mL) showed an absorbance peak at 275.7 nm. After the interaction of the fraction with Cu^2+^ ions, a red shift from 275.7 nm to 288.5 nm was confirmed. When it was treated with Zn^2+^ and Fe^2+^ ions, a red shift from 275.7 nm to 282.1 nm was also observed (Figure 5).

**Figure 6:**
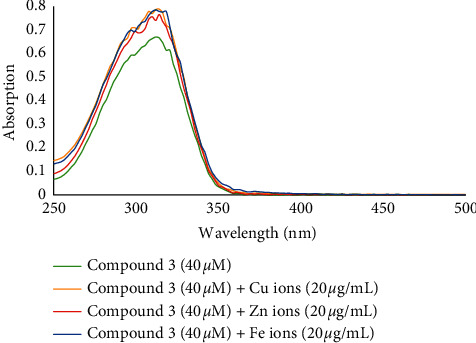
The absorbance changes of compound 3 alone and in the presence of Zn^2+^, Fe^2+^, and Cu^2+^ ions.

Investigation of metal chelating ability of isolated compounds **1**–**5** showed that only compound **3** (final concentration of 40 *μ*M) was able to form chelates with biometals (Figure 6). Interaction of compound **3** and Zn^2+^ and Cu^2+^ ions afforded two blue shifts from 320.5 and 312.0 nm to 314.1 and 307.7 nm, respectively. Moreover, the interaction of compound **3** and Fe^2+^ ions led to a change in the absorbance from 320.5 to 318.4 nm (Figure 6).

### 3.4. Antioxidant Activity by DPPH Assay

DPPH is a stable free radical that can accept an electron or hydrogen radical to become a stable molecule. All fractions (PE, DCM, EtOAc, and MeOH) of *L. inermis* were screened for its possible antioxidant activity by 2,2-diphenyl-1-picrylhydrazyl radical (DPPH) scavenging activity comparing with butylated hydroxyanisole (BHA) as the standard drug (IC_50_ = 3.79 ± 0.37 *μ*g/mL). As shown in Table 3, EtOAc and MeOH fractions were found to be the most potent antioxidant fractions (IC_50_ = 3.08 ± 0.10 *μ*g/mL and 3.79 ± 0.37 *μ*g/mL, respectively) as potent as the positive control. However, both PE and DCM fractions showed lower antioxidant activity than BHA.

### 3.5. Molecular Docking Study

To gain an insight into the binding interaction of the isolated compounds (**2** and **5**) with BChE (PDB ID: 4TPK), molecular docking experiments were performed using the Glide module (autodock suite). Docking scores in kJ/mol are shown in Table 4.

**Figure 7:**
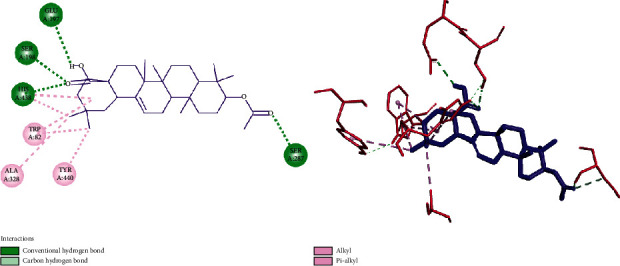
Two‐ and three‐dimensional representation of the docked pose of compound **2** into the binding pocket of the 4TPK.

As shown in Figure 7, compound **2** made desired interactions with the BChE active site. The carbonyl group of carboxylic acid established H-bonding interaction with Glu197, Ser198, and His438 residues. Carbonyl group of ester moiety formed a hydrogen bond with Ser287. Other interactions including alkyl and pi-alkyl interactions were constructed through the hydrophobic backbone of compound **2** and His438, Tr982, Ala328, and Tyr440 residues.

**Figure 8:**
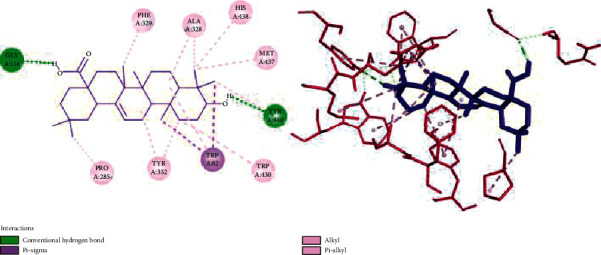
Two‐ and three‐dimensional representation of the docked pose of compound 5 into the binding pocket of the 4TPK.

In the case compound **5** (Figure 8), two hydroxyl groups formed two H-bonding interactions with Gly116 and Tyr440 residues of the BChE. Alkyl and pi-alkyl interactions were constructed between hydrocarbon moiety of compound **5** and Phe329, Ala328, His438, Met437, Trp430, Tyr332, Tyr440, Trp82, and Pro285. Moreover, two pi-sigma interactions were formed between methyl groups and Trp82.

## 4. Conclusion

In this study, different biological activities of *Lawsonia inermis* L. seeds related to AD as well as phytochemical analysis were investigated. *In vitro* anti-ChE activity of all fractions indicated potent and selective BChE inhibitory activity of the dichloromethane fraction which led to the isolation and identification of *β*-sitosterol (**1**), 3*-O-β-*acetyloleanolic acid (**2**), 3-*O*-(*Z*)-coumaroyl oleanolic acid (**3**), betulinic acid (**4**), and oleanolic acid (**5**). Evaluation of compounds **1**–**5** toward AChE and BChE revealed that compounds **2** and **5** were the most potent and selective inhibitors of BChE with IC_50_ values of 77.13 and 72.20 *μ*M, respectively. Furthermore, evaluation of the antioxidant and metal chelating ability of fractions and compounds confirmed the capability of *L. inermis* seeds to be considered in the treatment of AD.

## Figures and Tables

**Table 1:** ^13^C NMR data of isolated compounds **1**–**5** based on DEPT, HMQC, and HMBC experiments.

Position	*δ*_C_ (ppm)				
	1	2	3	4	5
1	37.3	38.1	37.4	38.4	38.0
2	31.7	23.5	23.3	27.1	27.1
3	71.8	80.9	79.7	76.7	76.8
4	42.3	37.7	37.5	38.2	38.3
5	140.8	55.3	54.5	54.8	54.7
6	121.7	18.2	17.8	17.9	18.0
7	31.9	32.5	32.0	33.8	32.0
8	31.9	39.3	38.8	40.2	38.8
9	50.1	47.5	46.8	49.8	47.0
10	36.5	37.0	36.5	36.3	36.5
11	21.1	22.9	22.8	20.4	22.8
12	39.8	122.6	121.4	25.0	121.5
13	42.2	143.6	144.5	37.5	143.8
14	56.8	41.5	41.3	41.9	40.7
15	26.1	27.6	27.2	30.0	26.9
16	28.3	23.4	22.6	31.6	22.0
17	56.1	46.5	45.6	55.4	45.6
18	11.9	40.9	40.8	46.6	41.2
19	19.4	45.8	45.4	48.4	45.4
20	36.2	30.7	30.4	150.3	29.0
21	18.8	33.8	33.3	29.1	33.2
22	33.9	32.4	32.2	36.7	32.8
23	26.1	28.0	27.8	28.0	28.2
24	45.1	15.4	15.0	15.7	16.0
25	29.2	16.7	16.7	15.9	15.0
26	19.1	17.2	16.8	15.8	16.8
27	19.8	25.9	25.5	14.3	25.5
28	23.1	183.8	178.6	177.3	178.6
29	12.0	33.1	32.8	18.9	30.3
30	—	23.6	23.3	109.7	23.3
CH_3_COO	—	21.3	—	—	—
CH_3_COO	—	171.1	—	—	—
1′	—	—	166.4	—	—
2′	—	—	114.5	—	—
3′	—	—	143.8	—	—
4′	—	—	125.0	—	—
5′	—	—	130.3	—	—
6′	—	—	115.7	—	—
7′	—	—	159.8	—	—
8′	—	—	115.7	—	—
9′	—	—	130.3	—	—

**Table 2:** AChE and BChE inhibitory activity of fractions and isolated compounds from *L. inermis*.

Fractions	AChE IC_50_ (*μ*g/mL)	BChE IC_50_ (*μ*g/mL)	Compounds	AChE IC_50_ (*μ*M)	BChE IC_50_ (*μ*M)
PE	>500	>500	**1**	>100	>100
DCM	>500	113.47 ± 1.25	**2**	>100	77.13 ± 0.01
EtOAc	>500	124.90 ± 1.15	**3**	>100	>100
MeOH	>500	>500	**4**	>100	>100
Donepezil	0.03 ± 0.00	1.52 ± 0.10	**5**	>100	72.20 ± 0.42
			Donepezil	0.08 ± 0.01	3.99 ± 0.27

^*∗*^Data are expressed as mean ± SD (three independent experiments).

**Table 3:** Free radical scavenging activities of the fractions of *L. inermis*.

Fraction	IC_50_ (*μ*g/mL)
PE	29.40 ± 1.61
DCM	23.45 ± 1.25
EtOAc	3.08 ± 0.10
MeOH	3.64 ± 0.03
BHA	3.79 ± 0.37

**Table 4:** Molecular docking analysis of isolated compounds from *L. inermis* in the active site of AChE and BChE.

Compound	Docking score (kJ/mol)
	BChE (PDB: 4TPK)
**2**	−9.06
**5**	−9.05
Donepezil	−9.53

## Data Availability

The datasets generated during and/or analyzed during the current study are available from the corresponding author on reasonable request.

## Abbreviations

*L. inermis*: *Lawsonia inermis*
AD: Alzheimer's disease
ACh: Acetylcholine
AChE: Acetylcholinesterase
AChEI: Acetylcholinesterase inhibitory
BChE: Butyrylcholinesterase
BChEI: Butyrylcholinesterase inhibitory
ChEs: Cholinesterases
DPPH: 2,2-diphenyl-1-picrylhydrazyl
IC_50_: The half maximal inhibitory concentration
BHA: Butylated hydroxyanisole
NFTs: Neurofibrillary tangles
ROS: Reactive oxygen species
FDA: Food and Drug Administration
ITM: Iranian Traditional Medicine
^1^H NMR: Proton nuclear magnetic resonance
^13^C NMR: Carbon-13 nuclear magnetic resonance
HMQC: Heteronuclear multiple quantum correlation
COSY: Correlation spectroscopy
HMBC: Heteronuclear multiple bond correlation
CC: Column chromatography
TLC: Thin-layer chromatography
UV: Ultraviolet radiation
ATCI: Acetylthiocholine iodide
DTNB: 5,5-Dithiobis-2-nitrobenzoic acid
MeOH: Methanol
EtOAc: Ethyl acetate
DCM: Dichloromethane
PE: Petroleum ether
ACE: Acetone.
